# LC–MS Metabolomic Profiling of Five Types of Unrefined, Cold-Pressed Seed Oils to Identify Markers to Determine Oil Authenticity and to Test for Oil Adulteration

**DOI:** 10.3390/molecules28124754

**Published:** 2023-06-14

**Authors:** Agata Sumara, Anna Stachniuk, Alicja Trzpil, Adrian Bartoszek, Magdalena Montowska, Emilia Fornal

**Affiliations:** 1 Department of Bioanalytics, Medical University of Lublin, ul. Jaczewskiego 8b, 20-090 Lublin, Poland; agata.sumara@umlub.pl (A.S.); anna.stachniuk@umlub.pl (A.S.); alicja.trzpil@umlub.pl (A.T.); adrian.bartoszek@umlub.pl (A.B.); 2 Department of Meat Technology, Poznan University of Life Sciences, ul. Wojska Polskiego 31, 60-624 Poznan, Poland; magdalena.montowska@up.poznan.pl

**Keywords:** plant oil, LC-MS, metabolomic, oil markers, authenticity testing, detection of adulteration

## Abstract

The authenticity of food products marketed as health-promoting foods—especially unrefined, cold-pressed seed oils—should be controlled to ensure their quality and safeguard consumers and patients. Metabolomic profiling using liquid chromatography coupled to quadrupole time-of-flight mass spectrometry (LC–QTOF) was employed to identify authenticity markers for five types of unrefined, cold-pressed seed oils: black seed oil (*Nigella sativa* L.), pumpkin seed oil (*Cucurbita pepo* L.), evening primrose oil (*Oenothera biennis* L.), hemp oil (*Cannabis sativa* L.) and milk thistle oil (*Silybum marianum*). Of the 36 oil-specific markers detected, 10 were established for black seed oil, 8 for evening primrose seed oil, 7 for hemp seed oil, 4 for milk thistle seed oil and 7 for pumpkin seed oil. In addition, the influence of matrix variability on the oil-specific metabolic markers was examined by studying binary oil mixtures containing varying volume percentages of each tested oil and each of three potential adulterants: sunflower, rapeseed and sesame oil. The presence of oil-specific markers was confirmed in 7 commercial oil mix products. The identified 36 oil-specific metabolic markers proved useful for confirming the authenticity of the five target seed oils. The ability to detect adulterations of these oils with sunflower, rapeseed and sesame oil was demonstrated.

## 1. Introduction

Lifestyle changes, such as medications, should be tailored to the individual. Lifestyle changes are based on four main pillars: nutrition, sleep, physical activity and stress management. The National Sleep Foundation recommends that an adult get from 7 to 9 h of sleep each night, whereas school-age children should get between 9 and 11 h. The quality of sleep can be improved by making use of the natural circadian rhythm. Furthermore, blue light emitted from the screens of electronic devices should be avoided before bedtime. Physical activity significantly reduces the risk of heart disease, enhances brain function and helps improve mood. Stress management focuses on reducing stress through socializing, spending time outdoors or taking hot, relaxing baths [1].

Nutrition, one of the most important pillars, should focus consistently on consuming healthy foods that provide adequate but not excessive calories. This helps to avoid becoming overweight or underweight and provides the body with the necessary minerals and vitamins [2]. Accordingly, highly processed foods should be avoided, sugar intake reduced, fruit and vegetable consumption increased and fiber-rich products, and those supporting a proper gut microbiome, should be central to the diet [1]. The education of consumers regarding these subjects would benefit from promoting traditional, regional food products grown on organic farms and learning from traditional medicine [2].

Seed oils have been used since antiquity not only as food but also as the first pharmaceuticals and cosmetics [3]. Seed oils are an important part of the diet because they contain fatty acids, including unsaturated fatty acids, such as omega-3 (α-linolenic acid) and omega-6 (linoleic acid) fatty acids, which are not synthesized by the human body and must thus be supplied by the daily diet. Fatty acids are necessary for the transmission of nerve impulses, the synthesis of hemoglobin and for cell division [4]. Seed oils are also rich in significant phytochemicals, such as phenolic compounds, sterols, carotenoids, minerals and vitamins, making these oils useful in the treatment of various diseases. Black seed oil (*Nigella sativa* L.) is used to treat type 2 diabetes [5,6], and evening primrose oil (*Oenothera biennis* L.) is used to treat mastalgia [7] and polycystic ovary syndrome [8]. Pumpkin seed oil (*Cucurbitapepo* L.) is used to treat overactive bladder [9] and chronic nonbacterial prostatitis [10]. Milk thistle oil (*Silybum marianum*) aids in the treatment of hepatic steatosis [11], and hemp oil (*Cannabis sativa* L.) helps treat atopic dermatitis [12]. However, seed oils used for these purposes must be produced from high-quality seeds and pressed using techniques that provide oil products that promote health and meet consumers’ expectations in terms of both quality and taste.

In an effort to provide consumers with the highest quality products, analytical methods have been developed to assess the purity of a product, confirm its authenticity or detect adulteration [13]. The adulteration of seed oils is not a challenge for producers, as adulterated and authentic products generally have the same consistency and similar colors, and oils are typically stored in dark bottles to protect them from sunlight. Several analytical methods have been developed to detect the adulteration of seed oils using specialized equipment and expert knowledge. For example, high-resolution mass spectrometry was used by Kotecka-Majchrzak et al. [14] to detect new species-specific peptide markers for 10 cold-pressed seed oils. Wei et al. [15] identified triacylglycerols (TAGs), which might provide a quick method for screening for oil adulteration using ion mobility spectroscopy [16] and total synchronous fluorescence spectroscopy (TSyF) [17]. Untargeted metabolomics coupled with high-resolution mass spectrometry allowed fingerprinting analysis and the identification of specific markers for detecting the adulteration of sesame oil [18]. Metabolomics has been applied in food research to assess food safety, food composition and food adulteration. The application of metabolomic analysis techniques was summarized by Wu et al. [19]. Furthermore, the use of LC-MS along with metabolic studies has contributed to the development of an analytical method to detect adulteration of cameline oil with soybean, peanut and canola oils. Additionally, metabolic studies were carried out to detect adulteration of olive oil with hazelnut oil. It is recommended that in order to confirm the authenticity of the oils or to detect their adulteration using metabolomic together with mass spectrometry larger number of markers is required since their occurrence or intensity may depend on the geographic conditions in which crops are grown [20,21].

In this work, we applied an untargeted approach combining chemometrics and high-performance liquid chromatography coupled with a quadrupole time-of-flight mass spectrometer (LC–QTOF) to detect and identify new oil-specific metabolic markers for five plant oils: black seed oil (*Nigella sativa* L.), pumpkin seed oil (*Cucurbita pepo* L.), evening primrose oil (*Oenothera biennis* L.), hemp oil (*Cannabis sativa* L.) and milk thistle oil (*Silybum marianum*). To study the effects of matrix variability on the stability, sensitivity and selectivity of the newly identified metabolic markers, binary oil mixtures of the oils analyzed in this study were prepared with three different oil matrices: sunflower oil (SO), rapeseed oil (RO) and sesame oil (SeO). The effect was evaluated by studying the linearity of the MS signals for oil-specific markers and the limits of detection for each marker were estimated.

## 2. Results and Discussion

### 2.1. Identification of Oil-Specific Metabolomic Markers

**Figure 1:**
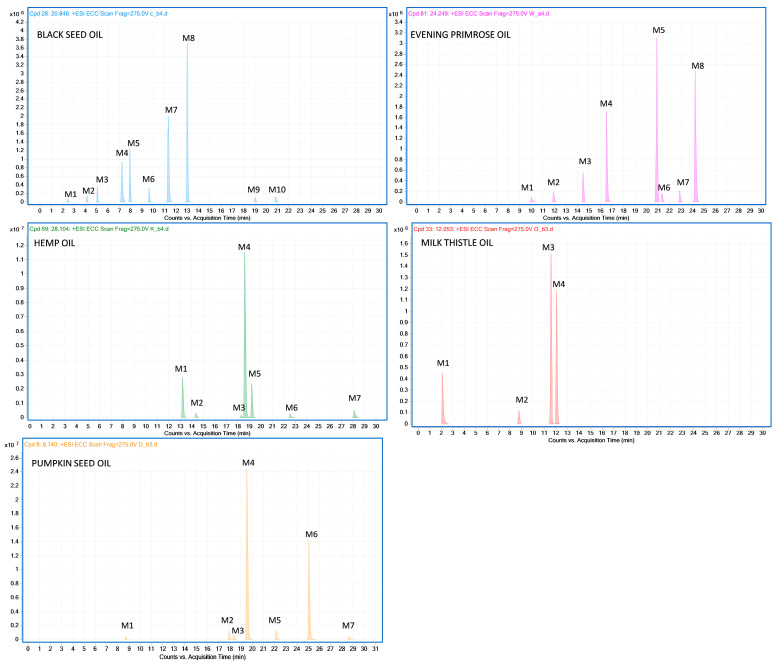
LC–QTOF extracted ion chromatograms of 10 oil-specific metabolomic markers of black seed oil (*Nigella sativa* L.), 8 oil-specific metabolomic markers of evening primrose oil (*Oenothera biennis* L.), 7 oil-specific metabolomic markers of hemp oil (*Cannabis sativa* L.), 4 oil-specific metabolomic markers of milk thistle oil (*Silybum marianum*) and 7 oil-specific metabolomic markers of pumpkin seed oil (*Cucurbita pepo* L.).

An untargeted metabolomic approach was conducted to identify characteristic metabolic features (ions of defined *m*/*z* and retention time) within the analyzed species. This approach provided information on known and unknown metabolites found in the analyzed oils. To select the markers that are characteristic of a given plant species, oils from nine species of plants (black seed, evening primrose, hemp, milk thistle, pumpkin seed, sunflower, rapeseed, flax and sesame oils) were used. We procured eight samples from four different producers for each species. The metabolomic marker finally selected as the oil-specific marker had to meet four requirements: be present in all eight samples of oil prepared from the same species, be absent in the remaining 64 samples of oils, be absent in the organic solvent used in sample extraction and be of high intensity. The list of selected oil-specific candidate compounds obtained during MPP filtering was verified manually to confirm their presence in the raw MS data across all oil samples of the given species using MassHunter Qualitative Analysis B.10.00 software (Agilent Technologies, Santa Clara, California, USA). Of the 229 compounds from black seed oil, 10 met all four of the above criteria, and the highest intensity ions were at *m*/*z* 404.2641 (M8) and *m*/*z* 385.2376 (M7) (Figure 1). For evening primrose oil, 207 compounds were initially obtained, 8 of which were identified as evening primrose oil-specific markers with the highest intensities for the ions at *m*/*z* 720.4547 (M5) and *m*/*z* 429.3741 (M7) (Figure 1). Of the 87 compounds from hemp oil, 7 markers were ultimately confirmed as hemp oil-specific, of which the most intense were the ions at *m*/*z* 341.2123 (M4) and *m*/*z* 375.2178 (M1) (Figure 1). Milk thistle oil analyses initially showed 295 compounds, but only 4 met the milk thistle oil-specific criteria, with the highest intensities for the ions at *m*/*z* 233.1545 (M3) and *m*/*z* 239.2013 (M4) (Figure 1). Of 274 compounds from pumpkin seed oil, we ultimately determined 7 as pumpkin seed oil-specific markers, of which the ions at *m*/*z* 578.4219 (M4) and *m*/*z* 682.4482 (M7) were the most intense (Figure 1). All 36 identified oil-specific metabolomic markers were then fragmented using targeted MS/MS QTOF analysis mode. The product spectra were acquired at collision energies in the range from 10 to 40 eV. Detailed fragmentation data are shown in Table 1.

### 2.2. Fragment Spectra Matching

**Figure 2:**
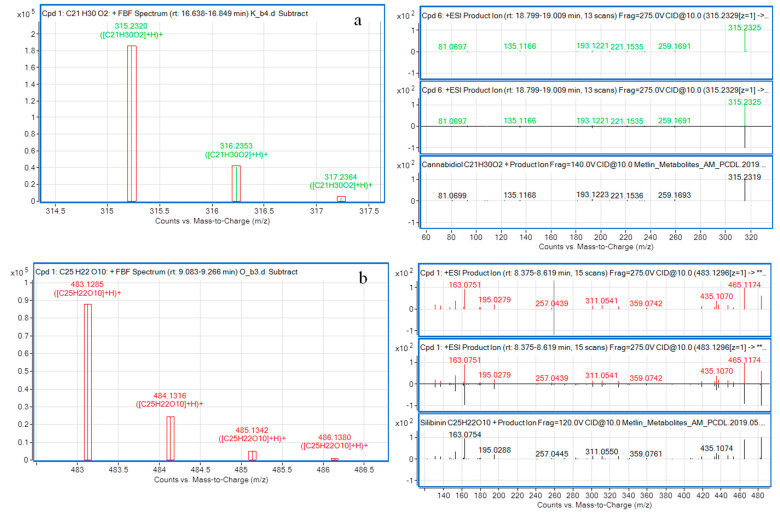
(**a**) Cannabidiol (hemp oil) and (**b**) silibinin (milk thistle oil) MS and MS/MS spectra; mirrored were identical to those in the METLIN PCDL (MS/MS spectra and isotope distributions).

The acquired MS/MS spectra of 36 oil-specific metabolomic markers were searched against MassHunter METLIN Metabolite Personal Compound Database and Library (Agilent Technologies, Santa Clara, CA, USA). METLIN is a database created in 2003 for tandem mass spectrometry and enables the identification of metabolites. The database stores data acquired in positive and negative ionization modes and multiple collision energy data from high resolution spectra. The METLIN database contains molecular standards, including metabolites, drugs and toxins [22]. The assignment of oil-specific metabolomic marker candidates to the measured signal was conducted by comparing the experimental spectra with the MS/MS spectrum available in the METLIN PCDL. A positive match was based on the MS accurate mass (mass error < 1 ppm, isotopic distribution score > 95%) and MS/MS spectral match (matching score > 95%) (Figure 2). We assigned two compounds. One is cannabidiol (CBD; C_21_H_30_O_2_), the compound with characteristic C21 terpeno-phenolic backbone present in plants from the Cannabaceae family and in hemp oil [23]. This compound is considered to be a potential drug as a palliative treatment or for combination therapy for cancer patients [24]. Citti et al. [25] identified CBD in 13 commercially available hemp oils at concentrations between 2.575 and 233.8 mg/kg. The second oil-specific marker candidate identified in the METLIN PCDL is silibinin (C_25_H_22_O_10_). This compound is a polyphenolic flavonoid antioxidant that makes up 50–60% of the silymarin complex. Silibinin has antioxidant properties and modulates insulin resistance [26]. These metabolites are commonly found in milk thistle oil (silibinin) and hemp oil (cannabidiol), and thus, they can be used as specific markers to confirm the authenticity of thistle oil and hemp oil. The measured MS/MS spectra of a cannabidiol candidate in hemp oil and a silibinin candidate in milk thistle oil were identical to the MS/MS spectra of cannabidiol and silibinin available in the METLIN PCDL, and the isotope distributions of the two are shown in Figure 2a,b, respectively.

### 2.3. Molecular Formula Assignment

LC–QTOF, which is characterized by high-resolution, allows the separation of ions with the same nominal weight but with a different molecular formula. For the remaining selected characteristic ions, for which we did not find a match in the METLIN PCDL, the formulas were generated using the compound algorithm in Agilent Mass Hunter Qualitative Analysis B.10.00 software (Agilent Technologies, Santa Clara, California, USA). The following parameters were applied: positive H^+^ ion with elements that may appear in formulas, including C, H, O, N, S and Cl; peak sparing tolerance 0.0025 *m*/*z*; mass differences 5 ppm; and the matching scores > 95%. The assigned formulas are reported in Table 1.

Schymanski et al. [27] conducted a literature review and proposed a 5 level system of small molecule identification by liquid chromatography—high resolution mass spectrometry (LC–HRMS). Level 1—confirmed structure: confirmation of the compound using the reference standard, from the MS, MS/MS level and retention time. Level 2—probable: identification of the compound by matching information from the literature or library spectral data. Level 3—tentative candidate: assumptions are required to confirm the structure, so there is no definitive confirmation. Level 4—unequivocal molecule: generates a pattern using the interpretation of observed *m*/*z*, adducts, isotope patterns and fragment information. Level 5—exact mass: a compound referred to as “unknown”; observed *m*/*z* and MS/MS fragments are the only known information.

Thirty-six specific markers were selected as authenticity markers for five types of unrefined, cold-pressed seed oils. Two were identified at level 2 according to the classification proposed by Schymanski et al. [27]: silibinin in milk thistle oil and cannabidiol in hemp oil. A further 31 markers were identified at level 4, and 3 at level 5 (Table 1). A similar interpretation of the results was made by Cavanna et al. [28], who used LC–HRMS to select metabolic markers to detect soft-refined oil additives to extra virgin olive oils. They identified 12 molecules, 2 of which were identified at level 2, 6 at level 3 and 4 at level 4. Cao et al. [29] used an untargeted and pseudotargeted approach to indicate potential markers differentiating live pigs from pork meat and detected 24 metabolite markers. The identification of markers was carried out using the METLIN database and reference standards. They identified two markers with reference standards, for next six markers they found match in the database. For other six markers, they predicted formulas with mass error < 5 ppm (level 4). The remaining seven of the 24 markers were marked at level 5. Moreover, Garcia et al. [30] also used the LC–MS untargeted metabolomic approach to predict the browning of fresh-cut lettuce, which significantly decreases the quality of the vegetable. They used statistical software, allowing the selection of a group of metabolites. Several of the selected metabolites were confirmed using reference standards. Unknown metabolites, identified at levels 4 and 5, were also reported.

### 2.4. Effect of Matrix Variability on Marker Signal

To study the effects of matrix variability on the stability, sensitivity and selectivity of 36 oil-specific metabolomic markers, binary mixtures of the 5 oils analyzed in this study and 3 different oil matrices (SO, RO and SeO) were prepared. These oils were chosen for the two-component mixtures because they are several times cheaper than the oils tested. Sunflower and rapeseed oils are often identified as cheap additives, as they lower the final cost of production [31].

The correlation between the peak area of the 36 oil-specific metabolomic markers and the percentage concentration of the five oils selected in this study was evaluated in the range of 5–90% (*v*/*v*). Of the ten metabolomic markers for black seed oil, seven showed acceptable linearity (R^2^ > 0.95) measured in all tested oil matrices; for evening primrose oil, all eight markers met the set requirements. Of the seven metabolomic markers selected for hemp oil, only one marker (*m/z* 375.2178 (M1)) was found with an R^2^ value over 0.95 in all tested oil matrices, whereas three markers (*m/z* 369.1343 (M2), *m/z* 437.1965 (M3), *m/z* 341.2123 (M4)) met these requirements in mixtures with SO and RO. Of the four metabolomic markers selected for milk thistle oil, three (*m/z* 483.1296 (M2), *m/z* 233.1545(M3), *m/z* 239.2013 (M4)) showed adequate R^2^ values for all tested oil matrices. All metabolomic markers for pumpkin seed oil showed good linearity, with R^2^ coefficients exceeding 0.95. The findings indicate that that the change of sample composition resulting from adding adulterant oil does not negatively affect marker selectivity and detectability, and thus their utility.

In the next step, we calculated the LOD for the metabolomic markers that met the requirement of R^2^ > 0.95. Of the selected markers for black seed oil, five were characterized by very low detection limits and low signal variability and are supremely good candidates for routine oil authentication. The ranges of LOD values were as follows: 0.06–0.16% [*v*/*v*] (M8, *m/z* = 404.2641); 0.07–0.29% [*v*/*v*] (M7, *m/z* = 385.2376); 0.58–0.84% [*v*/*v*] (M4, *m/z* = 313.1802); 0.29–0.38% [*v*/*v*] (M5, *m/z* = 238.0901); and 0.94–1.53% [*v*/*v*] (M1, *m/z* = 166.1226), respectively. Given that the diversity and complexity of the matrix of the analyzed sample may affect the intensity or attenuation of the signal of the analyzed ion, the calculated LOD values also demonstrate the stability and sensitivity of the characteristic markers in relation to changing the matrix of the analyzed sample. All eight metabolomic ion markers for evening primrose oil met the requirements for R^2^. The lowest LOD values were for marker M4, *m/z* = 492.3174, in the range of 0.13–0.21% [*v*/*v*] and the highest LOD values were for marker M7, *m/z* = 577.3900, in the range of 3.83–4.19% [*v*/*v*]. Of the markers selected for hemp oil, only marker M1, *m/z* = 375.2178, with an LOD of 0.2–0.33% [*v*/*v*] had R^2^ > 0.95. For cannabidiol (M5, *m/z* = 315.2319), the values of LOD were not calculated because the R^2^ coefficients did not exceed 0.95. Of the markers selected for milk thistle oil, three met the requirements for R^2^. The LOD values for these markers were in the following ranges: 0.24–0.8% [*v*/*v*] (M3, *m/z* = 233.1545); 0.31–0.7% (M4, *m/z* = 239.2013); and 2.11–2.22% [*v*/*v*] (M2, *m/z* = 483.1286, silibinin), respectively. Rahal et al. [32] determined the content of silibinin in milk thistle oil using supercritical carbon dioxide to be in the range of 51.1 ± 1.3 to 140.24 ± 4.99 µg/mL; however, they did not determine the LOD value. All metabolomic markers for pumpkin seed oil (M1–M7) had R^2^ values of > 0.95. The LOD range for the highest intensity marker (M4, *m/z* = 578.4219) was 0.03–0.12%. All LOD results are presented in Table 2. The obtained LOD results enabled us to detect the presence of individual oils in products, even if their content was < 5%. This is extremely important in economic terms, as the oils analyzed in this study are often used as ingredients in cosmetics in low concentrations owing to their high price. In addition, the LOD calculations for two-component mixtures for five studied oils showed that most of the ions, for which these values were calculated, are stable, regardless of the matrix of the analyzed sample.

### 2.5. Variability in Marker Intensity

The oil plants from which seed oils are pressed can be grown under a wide variety of conditions; their quality is affected by sunlight, humidity or soil type, and other factors, and thus, the final oil products may vary. To assess differences in products from various producers and between production batches, we evaluated the variability of intensity of species–specific markers by calculating the percent relative standard deviation (RSD) of marker EIC peak areas. For black seed oil, marker M1 (*m/z* 166.1226) showed the smallest difference in intensity, with an RSD of 36.48%, whereas the highest was for the marker M8, *m/z* 404.2641, where the value was 128.73%. The RSD for hemp oil was in the range of 46–65.6%. For milk thistle oil, all markers showed an RSD above 60%. For evening primrose oil, the four markers analyzed did not exceed an RSD of 31%, although the highest value was observed for the M6 marker (*m/z* 720.4547, 92%). Of the seven markers for pumpkin seed oil, the lowest variability was observed for marker M4, *m/z* 578.4219 (14.72%), and the highest was for M5, *m/z* 697.4592, at 66.8%. The RSD values for the other markers are shown in Table S2 in the Supplementary Materials. Despite the different intensities of the markers selected from five types of seed oils, these markers can be effectively used in the qualitative analyses for oils authentication and adulteration testing. Their utility for quantitative analyses seems to be limited, as the uncertainty of the determination would be high.

### 2.6. Detection of Sunflower, Sesame and Rapeseed Oils in Binary Mixtures

**Figure 3:**
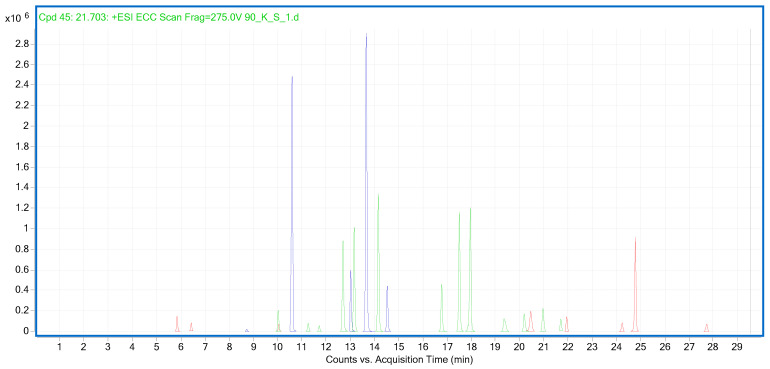
LC–QTOF–MS extracted ion chromatograms of 13 specific markers for sunflower oil (green), 8 markers for rapeseed oil (red) and 5 markers for sesame oil (blue) detected in binary oil mixtures at a level of 10% *v*/*v* (see Table S3 for details).

**Figure 4:**
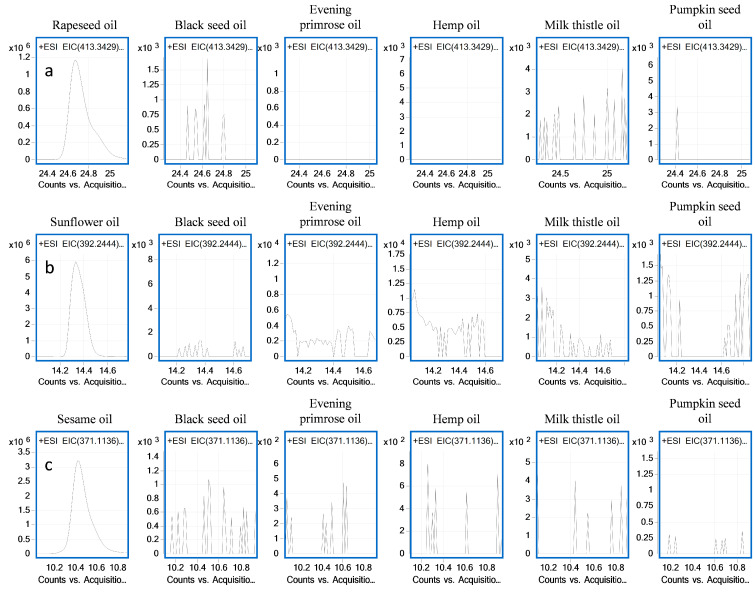
LC–QTOF–MS-level extracted ion chromatograms of the metabolite markers for (**a**) rapeseed oil *m/z* 413.3429, (**b**) sunflower oil *m/z* 392.2444 and (**c**) sesame oil *m/z* 371.1136 from binary mixture samples and their comparison with black seed, evening primrose, hemp, milk thistle and pumpkin seed oil samples.

In the next step of our study, we determined the presence of sunflower, sesame and rapeseed oils in the prepared binary oil mixtures using a list of previously published markers [33]. Owing to the higher production costs incurred by an edible oil manufacturer in the production of a high-quality product, such products are often adulterated using cheaper oils. In our previous study, to enable detection of such adulterations, we selected 13 markers specific to sunflower oil, 8 for rapeseed oil and 5 for sesame oil [33]. All these markers were detectable in binary mixtures containing 10% sunflower, rapeseed and sesame oils, and their LOD values were calculated. The lowest LOD values calculated for mixtures with rapeseed oil were 1.23–4.35% [*v*/*v*] and 0.15–4.35% [*v*/*v*] for ions *m/z* 162.056 and *m/z* 413.343, respectively. The LOD values calculated for sesame oil were 0.12–1.52% [*v*/*v*] and 0.11–3.03% [*v*/*v*] for markers *m/z* 371.1136 and *m/z* 173.0604, respectively. Markers for sunflower oil with good sensitivity potential include these of *m/z* 203.1076 and *m/z* 392.2444, with LOD values of 0.4–3.3% and [*v*/*v*] and 0.42–2.83% [*v*/*v*], respectively (Table S3). Figure 3 shows LC–QTOF–MS extracted ion chromatograms of 13 specific markers for sunflower oil, 8 markers for rapeseed oil and 5 markers for sesame oil detected in binary oil mixtures at a level of 10% *v*/*v*. None of these markers were detected in the oils included in the current study. The specificity of the markers is shown in Figure 4. These markers provide a tool for the qualitative verification of oil authenticity and for detecting adulteration, which is extremely important not only in economic terms but also with regard to the health benefits and risks of a particular oil.

### 2.7. The Detection of Specific Metabolite Markers in Commercial Unrefined, Cold-Pressed Oil Blends

In the next step of our study, we used selected metabolic markers to confirm the authenticity of commercial, unrefined, cold-pressed oil blends of two, three and four components. The purchased oils differed in both composition and proportions which, according to the manufacturer’s declaration, were specifically selected for particular target groups. The following blends were subject to analysis: oil for children (Mix1), oil for the heart (Mix 2), oil for women (Mix 3), oil for mothers (Mix 4), oil for elderly (Mix 5), oil for men (Mix 6) and immune oil (Mix 7). The composition of the products is listed in Table S4 in Supplementary Materials. We verified plant species compliance with oil labeling information by monitoring 36 species-specific markers identified in this study. The list was extended by 13 species-specific markers for sunflower oil and 3 species-specific flax seed oil markers from our previous study [33]. The results of the composition verification of 7 blends subject to analysis was presented in Table 3. The intensity of detected markers was shown on chromatograms in Figure S1 in Supplementary Materials. The presence of all 10 specific markers was confirmed for black seed oil, which was a component of Mix 1 (2%), Mix 7 (4%) and Mix 2 (20%). Evening primrose oil was the component of Mix 3 (3%), Mix 7 (5%) and Mix 4 (20%). Seven of eight metabolite markers for this oil were present in Mix 4, six of eight were detected in Mix 7, but only markers M1 (*m/z* 469.3317) and M7 (*m/z* 577.39) were found in the product with 3% contents of evening primrose oil. In the oil for men (Mix 6) we detected four of seven species-specific markers for hemp oil. The highest intensity was observed for M1 (*m*/*z* 375.2178) and M4 (*m*/*z* 341.2123). The presence of milk thistle oil in Mix 3 (27%) and Mix 5 (20%) was confirmed with all specific markers for the species. Pumpkin seed oil was in the composition of Mix 6 (10%) and Mix 7 (4%). In Mix 6, we detected six of 7 specific markers and in Mix 7 we found five of them. In both products, the M7 marker (*m/z* 578.5152) was not found. All 13 and 3 metabolites were detected in Mix 4 for sunflower oil and flaxseed oil, respectively. Moreover, all three markers for flaxseed oil were present in Mix 1, Mix 2, Mix 3, Mix 5 and Mix 6. The detection of the markers allowed the qualitative determination of the composition of commercially available oil blends and the confirmation of their authenticity. The markers that have been identified can be successfully used in screening, which, thanks to rapid analysis, allows the qualitative confirmation of the composition of the oil and oil blends where doubts may be raised as to their authenticity.

## 3. Materials and Methods

### 3.1. Materials

Acetonitrile and methanol (Optima^®^ LC-MS grade) were supplied by Fisher Chemical (Waltham, MA, USA). Formic acid (LC-MS grade) was obtained from Merck KGaA (Darmstadt, Germany). Extracts were filtered through a titan syringe filter (0.20 μm, 4 mm) from ThermoScientific (Lafayette, LA, USA). Ultrapure water was obtained using a Millipore Direct-Q3-UV purification system (Merck KGaA).

### 3.2. Samples

A total of 40 different cold-pressed seed oil samples of black seed oil (*Nigella sativa* L.), pumpkin seed oil (*Cucurbita pepo* L.), evening primrose oil (*Oenothera biennis* L.), hemp oil (*Cannabis sativa* L.) and milk thistle oil (*Silybum marianum*) were obtained. Sunflower (*Helianthus* L.), rapeseed (*Brassica napus* L.) and sesame (*Sesamum* L.) oils were purchased to prepare the binary oil mixtures, and flax seed oil (*Linum L.*) was used to verify marker specificity. Seven commercial unrefined, cold-pressed oil blend products were purchased to validate markers (Table S4). In total, 79 unrefined, cold-pressed seed oils from different Polish producers were used in this research. All bottles were stored in a refrigerator at 4 °C in their original bottles until use.

### 3.3. Preparation of the Oil Mixtures

Binary oil mixtures containing 5%, 10%, 30%, 50%, 70% and 90% [*v*/*v*] of black seed oil, pumpkin seed oil, evening primrose oil, hemp oil or milk thistle oil in sunflower oil (SO), rapeseed (RO) or sesame oil (SeO) were prepared (Supplementary Material Table S1). Each mixture was prepared in triplicate. In total, 270 samples were obtained. Before extraction, all binary oil mixtures were vortexed for 1 min.

### 3.4. Extraction

The oil samples and binary oil mixtures were prepared by liquid-liquid extraction. Into 0.5 mL of oil or mixture, 0.5 mL methanol-water was added (80:20, *v*/*v*). The samples were shaken by hand for 30 s and then vortexed for 2 min. The samples were then centrifuged for 5 min at 12,100× *g*, and 0.3 mL of the top layer was collected and mixed with 0.45 mL of ultrapure water. The samples were stored in a refrigerator at 4 °C for 15 min and then centrifuged for 30 min at 12,100 g. The top layer was collected and filtered through a titan syringe filter. The obtained samples were used for LC-MS analysis.

### 3.5. LC–QTOF Analysis

Metabolites were chromatographically separated on a 1290 Infinity high-performance liquid chromatograph (Agilent Technologies, Santa Clara, CA, USA) equipped with an RRHT Zorbax Extend C18 column (2.1 × 100 mm, 1.8 µm, Agilent Technologies). The mobile phase consisted of 0.1% formic acid in water (A) and 0.1% formic acid in acetonitrile (B) and was pumped at a flow rate of 0.4 mL/min. The gradient program was 0–25 min, 3%B to 95%B; 25–30 min, 95%B. The injection volume was 5 µL and the column temperature was 45 °C. Mass spectrometry analysis was performed on a 6550 iFunnel QTOF mass spectrometer (Agilent Technologies, Santa Clara, CA, USA) equipped with a Jet Stream Technology ion source operating in positive electrospray ionization mode (ESI+). The operation parameters were as follows: ion source gas (nitrogen) temperature: 225 °C; nitrogen flow rate: 12 L/min; nebulizer pressure: 50 psi; sheath gas temperature: 275 °C; sheath gas flow: 12 L/min and capillary voltage: 3500 V. The nozzle voltage was set at 1000 V and the fragmentor voltage at 275 V. The mass spectrometer was operated in MS scan mode at 1.2 spectra/s, and in targeted MS/MS mode at an MS scan rate of 8 spectra/s and MS/MS scan rate of 4 spectra/s. The product spectra were collected at three collision energies: 10, 20 and 40 eV. Internal mass calibration was enabled by using two reference masses, at 121.0509 and 922.0098 *m/z.* The LC–QTOF was controlled by Agilent Mass Hunter Data Acquisition B.09.00 software (Agilent Technologies, Santa Clara, California, USA. Data were processed using Agilent Mass Hunter Qualitative Analysis B.10.00 software (Agilent Technologies, Santa Clara, CA, USA.

### 3.6. LC–QTOF Data Processing

Oil-specific metabolomic markers for five seed oils were identified using bioinformatic tools. The analyses were performed on raw LC–MS data using the Agilent Mass Hunter Qualitative Analysis B.10.00 software (Agilent Technologies, Santa Clara, California, USA. The molecular features present in all analyzed samples were extracted using the following parameters: small molecules (chromatographic) as a target data type, background peak height set to 600 counts, absolute count for compound set to 50.000 counts, quality score ≥ 80, only H^+^ adduct, peak specific tolerance set to 0.0025 *m/z* plus 0.7 ppm and common organic molecules as an isotope model. All compounds (metabolomic features) meeting these requirements were converted to CEF files. The files were imported into Mass Profiler Professional (MPP) 15.1 software (Agilent Technologies, Santa Clara, California, USA) and further analyzed. All compounds were aligned with parameters of 0.1% + 0.15 min RT window and 5 ppm + 2.0 mDa mass window.

### 3.7. Evaluation of the Effect of Matrix Variability on Marker Signal

The effect of matrix variability on MS signals of oil-specific markers was evaluated by studying signal linearity. Linearity studies were based on the peak areas of the extracted ion chromatograms (EIC) for the 36 oil-specific metabolomic markers. Calibration curves were obtained in triplicate for five oils in three different oil combinations. Calibration curves were prepared for eight, seven, four and seven metabolomic markers for evening primrose oil, pumpkin seed oil, milk thistle oil and hemp oil, respectively. Linearity was assessed by comparing correlation coefficients (R^2^) and by analyzing the residuals. The acceptance criterion for correlation coefficients (R^2^) was set at 0.95. The LOD was determined for metabolic markers that met the criterion for linearity and were calculated as signal-to-noise ratios of 3.

## 4. Conclusions

An untargeted metabolomic approach using high-performance liquid chromatography coupled with high-resolution mass spectrometry was used to discover oil-specific metabolomic markers for five types of unrefined, cold-pressed seed oils. We identified a set of 36 metabolic markers: 10 for black seed oil, 8 for evening primrose oil, 7 for pumpkin seed oil, 4 for milk thistle oil and 7 for hemp oil which can be applied to the authentication of oils. The analytical method allowing detection of oil-specific markers is efficient, easy and fast. It requires only a simple liquid–liquid extraction process for sample preparation and a 30 min analytical run. Small quantities of chemicals are needed, the workload and costs of analysis are low. Simultaneously, in the same chromatographic run, the adulterations with other oils may be detected by monitoring markers of potential adulterant oils. Multiple reaction monitoring methods can also be developed for identified markers, thus further increasing the facility of method implementations in food control laboratories for routine verification of the composition of cold-pressed seed oils and rapid screening for oil adulterations.

## Figures and Tables

**Table 1:** Metabolomic markers of black seed oil, evening primrose oil, hemp oil, milk thistle oil and pumpkin seed oil detected by LC–QTOF.

Oil	No	Observed *m/z*	tR [min]	MS/MS [*m/z*]	CE [eV]	Generated Formula	Theoretical (*m/z*)	Score	Diff (ppm)
Black seed	M1	166.1226	2.6	124.0754, 123.0677, 106.0650, 109.0520, 91.0540	20	C10H15NO	166.1226	98.18	-
	M2	328.1551	4.2	297.1116, 265.0856, 237.0909, 266.0900	20	C17H19N4O3	328.1530	99.17	−1.06
	M3	342.1710	5.1	311.1279, 280.1095, 296.1042, 312.1306	10	C20H23NO4	342.1700	99.77	−0.52
	M4	313.1802	7.3	135.0803, 99.0440, 255.1347, 295.1639,71.0484	15	C20H24O3	313.1798	98.89	1.62
	M5	238.0901	7.9	220.0790, 205.0551, 192.0474, 187.0988,179.0310	15	C12H15NO2S	238.0896	99.2	−1.31
	M6	131.0862	9.7	91.0544, 115.0539, 65.0387, 128.0620, 77.0389	10	*	*	*	*
	M7	385.2376	11.4	231.1014, 367.2270, 273.1118, 203.1068, 229.1216	10	C22H30N3O3	385.236	99.86	−0.33
	M8	404.2641	13.1	207.1744, 85.0650, 133.1012, 225.1849, 189.1635	10	C20H37NO7	404.2643	99.63	0.71
	M9	677.3231	19.1	554.2903, 283.2053, 124.0393, 265.1937, 255.2086	10	C41H44N2O7	677.3221	99.52	0.61
	M10	635.3118	20.9	512.2796, 267.2105, 124.0391, 390.2424, 258.2207	10	C37H40N5O5	635.3102	99.22	−0.98
Evening Primrose	M1	469.3317	10.9	405.3150, 187.1477, 201.1632, 199.1480, 451.3206	20	C30H44O4	469.3312	96.56	0.74
	M2	455.3161	11.8	391.2988, 187.1471, 161.1317, 189.1269,191.1784	20	C29H42O4	455.3156	98.27	0.22
	M3	409.2207	14.4	163.0723, 55.0540, 269.1347, 69.0699, 112.9887	40	C18H28N6O5	409.2194	98.42	−1.79
	M4	492.3174	16.5	97.0647, 343.2116, 229.1432, 475.2904, 361.2220	40	C25H41N5O5	942.318	98.7	0.74
	M5	634.4167	20.9	617.3896, 503.3217, 97.0646, 211.1325, 485.3110	10	C33H55N5O7	634.4174	99.17	−0.03
	M6	720.4547	21.1	703.4265, 211.1325, 457.2794, 589.3585, 97.0647	10	*	*	*	*
	M7	577.39	22.9	409.3461, 203.1790, 437.3413, 205.1578, 425.3411	20	C37H52O5	577.3888	96.91	−0.31
	M8	429.3741	24.2	97.3727, 109.0646, 411.3619, 83.0852, 69.0699	20	C29H48O2	429.3727	97.88	−2.08
Hemp	M1	375.2178	13.3	357.2059, 339.1949, 275.1273, 358.2087, 340.1989	10	C22H30O5	375.2166	98.81	−1.57
	M2	369.1343	14.4	313.0703, 314.0739, 298.0458, 81.0692, 109.1004	20	C21H20O6	369.1333	97.56	−1.61
	M3	437.1965	18.3	313.0708, 350.1531, 89.0592, 396.1594, 320.1490	10	C26H28O6	437.1959	99.76	−0.24
	M4	341.2123	18.6	219.1014, 2 61.1483, 285.1481, 233.1168, 109.1009	20	C22H28O3	341.2111	98.03	−1.91
	M5	315.2329	19.3	193.1221, 135.1166, 259.1689, 93.0695, 81.0696	20	C21H30O2	315.2319	98.71	−1.75
	M6	498.3586	22.6	147.0439, 236.1272, 352.3204, 218.1185, 100.1115	10	*	*	*	*
	M7	460.4163	28.2	121.0646, 138.0912, 138.0912, 57.0699, 71.0854	20	C30H53NO2	460.4149	98.56	−0.79
Milk Thistle	M1	145.0764	2.1	78.0336, 117.0569, 51.0230, 91.0540, 65.0385	40	C9H8N2	145.076	99.52	−1.04
	M2	483.1296	8.8	153.0178, 195.0286, 131.0488, 163.0751, 437.1226	20	C25H22O10	483.1286	99.63	0.36
	M3	233.1545	11.6	95.0491, 135.0803, 175.1113, 215.1427, 107.0852	10	C15H20O2	233.1536	97.89	−1.71
	M4	239.2013	12.1	221.1902, 69.0701, 111.1167, 149.1321, 203.1792	10	C15H26O2	239.2006	99.44	−1.28
Pumpkin seed	M1	269.1904	8.7	195.1163, 185.1318, 145.0851, 199.1471, 227.1795	20	C19H24O	269.19	99.69	−0.37
	M2	181.1231	17.9	125.0593, 98.9609, 93.0695, 135.1165, 107.0852	20	C11H16O2	181.1223	98.57	−2.43
	M3	358.2389	18.4	137.0956, 312.2321, 218.1538, 93.0693, 44.0497	40	C22H31NO3	358.2377	98.74	−1.42
	M4	578.4219	19.6	405.3522, 423.3628, 120.0444, 231.1745, 138.0548	20	C38H51N5	578.4217	98.35	0.4
	M5	697.4592	22.1	405.3517, 423.3620, 542.3993, 120.0443, 560.4096	10	C45H56N6O	697.4588	98.84	−0.87
	M6	682.4482	25.1	527.3866, 405.3510, 423.3615, 189.1636, 59.00597	10	C45H55N5O	682.4479	99.05	0.27
	M7	578.5152	28.6	73.0464, 355.0709, 211.0850, 405.3460, 133.0843	20	C36H67NO4	578.5143	98.84	−0.87

No—number of metabolite marker (see Figure 1); tR—retention time; CE—collision energy; Diff—relative mass difference; Score—reflects how well the mass, isotope pattern and formula match the MS data; asterix (*)—no match, with score > 95%.

**Table 2:** Linearity parameters, LOD results for 36 oil-specific metabolomic ion markers.

No.	*m/z* [M + H]^+^	Regression Equation			R^2^			LOD [%, *v*/*v*]		
		SO	RO	SeO	SO	RO	SeO	SO	RO	SeO
				Black seed oil						
M1	166.1226	y = 789,870x + 72,825	y = 765,452x + 59,749	y = 753,902x + 43,066	0.9787	0.9707	0.9964	0.94	1.24	1.53
M2	328.1551	y = 40,413x + 1019	y = 31,236x − 122	y = 26,190x + 638	0.9972	0.993	0.999	8.82	20	12.5
M3	342.1710	y = 81,215x + 2180	y = 75,462x + 988	y = 66,784x + 861	0.9964	0.9982	0.9956	5.56	8.82	9.38
M4	313.1802	y = 5,478,074x + 285,848	y = 5,130,947x + 378,506	y = 5,125,274x + 302,426	0.9916	0.9855	0.9863	0.69	0.58	0.84
M5	238.0901	y = 14,043,686x + 157,530	y = 12,914,264x + 380,895	y = 12,737,396x + 225,908	0.999	0.9975	0.9983	0.38	0.29	0.36
M6	131.0862	y = 3,604,491x + 304,169	y = 1,540,440x + 422,467	y = 2,133,072x + 450,678	0.9756	0.9092	0.8801	6.52	*	*
M7	385.2376	y = 10,479,323x − 32,847	y = 10,368,286x + 64,006	y = 9,301,967x − 53,713	0.9961	0.9991	0.9951	0.29	0.08	0.07
M8	404.2641	y = 12,090,222x + 632,055	y = 11,022,281x + 629,354	y = 10,433,967x + 431,133	0.9938	0.9893	0.9952	0.14	0.07	0.16
M9	677.3231	y = 464,7476x − 461,806	y = 1,417,653x − 114,829	y = 1,819,364x − 269,027	0.9817	0.7397	0.8573	*	*	*
M10	635.3118	y = 6,161,239x − 457,510	y = 2,523,982x − 208,112	y = 2,934,313x − 392,763	0.9912	0.8349	0.9008	*	*	*
				Evening primrose oil						
M1	469.3317	y = 3,699,312x + 58,459	y = 4,493,887x + 71,465	y = 3,495,036x + 79,104	0.9996	0.9966	0.996	1.40	0.60	0.49
M2	455.3161	y = 297,109x + 9990	y = 348,873x + 3114	y = 302,159x + 2860	0.9982	0.9981	0.9937	3.95	1.67	1.22
M3	409.2207	y = 1,281,896x + 11305	y = 1,311,303x − 27,405	y = 1,499,518x + 21,161	0.9994	0.997	0.9959	0.7	0.74	0.75
M4	492.3174	y = 3,156,330x − 10739	y = 3,195,110x – 31,464	y = 3,224,260x + 19,978	0.9989	0.9996	0.9976	0.21	0.13	0.15
M5	634.4167	y = 2,937,485x + 53544	y = 3,762,151x + 23,698	y = 3,787,857x + 20,847	0.9922	0.9928	0.991	0.31	0.29	0.14
M6	720.4547	y = 2,677,027x − 15,907	y = 3,927,701x − 65,507	y = 3,951,119x − 21,469	0.9933	0.999	0.9897	0.19	0.18	0.21
M7	577.39	y = 221,915x + 11,190	y = 217,440x + 19,556	y = 261,795x + 2944	0.9958	0.9811	0.9951	4.05	3.85	4.19
M8	429.3741	y = 1,953,980x + 125,896	y = 2,251,678x + 81,880	y = 2,432,167x + 30,767	0.9885	0.9969	0.997	0.68	0.74	0.68
				Hemp oil						
M1	375.2178	y = 11,502,419x + 862,052	y = 12,203,972x + 4083	y = 1,170,168x + 1028.54	0.9851	0.993	0.9989	0.21	0.32	0.32
M2	369.1343	y = 833,660x + 34,874	y = 1,346,765x + 424	y = 1,155,917x − 50,071	0.9935	0.9921	0.947	0.5	2.5	*
M3	437.1965	y = 320,534x + 21,664	y = 757,857x + 35704	y = 562,753x + 27,474	0.0954	0.9939	0.8857	0.49	0.9	*
M4	341.2123	y = 3,134,659x + 3,220,487	y = 5,694,353x + 5699612	y = 5,155,590x + 7,664,520	0.9398	0.9502	0.8821	*	0.04	*
M5	315.2329	y = 5,627,335x + 238,675	y = 14,455,766x + 38848	y = 11,885,033x − 564,670	0.9368	0.9946	0.8898	*	0.1	*
M6	498.3586	y = 404,963x + 23,137	y = 1,264,650x + 33514	y = 833,971x − 13,357	0.9345	0.9938	0.8757	*	0.4	*
M7	460.4163	y = 1,214,547x + 25,860	y = 2,336,849x + 2926	y = 1,558,213x − 20893	0.9879	0.9988	0.892	0.23	0.80	*
				Milk thistle oil						
M1	145.0764	y = 630,817x − 7184	y = 441,295x + 16,516	y = 304,454x + 37,802	0.9342	0.9892	0.9399	*	2.27	*
M2	483.1296	y = 217,321x − 2053	y = 203,452x + 3123	y = 195,280x + 2726	0.9931	0.9959	0.9860	2.22	2.16	2.11
M3	233.1545	y = 4,130,535x + 175,405	y = 3,660,480x + 120,505	y = 3,229,384x + 123,499	0.997	0.9987	0.9942	0.8	0.24	0.35
M4	239.2013	y = 2,117,856x + 316,00	y = 1,848,032x + 34,660	y = 1,710,753x + 51,351	0.9979	0.9989	0.9917	0.67	0.31	0.5
				Pumpkin seed oil						
M1	269.1904	y = 690,664x − 3950	y = 627,498x − 5341	y = 676,594x − 8551	0.9985	0.997	0.9958	1.1	1.42	0.64
M2	181.1231	y = 2,516,933x − 46,852	y = 2,998,961x + 109,634	y = 2,628,907x − 34,395	0.9973	0.9973	0.9985	0.7	0.19	0.70
M3	358.2389	y = 1,112,206x − 19,675	y = 1,665,518x − 9513	y = 1,130,042x − 61,697	0.9986	0.9974	0.9922	0.34	0.22	0.22
M4	578.4219	y = 26,872,797x − 123,014	y = 30,525,005x − 233,735	y = 24,765,411x − 166,352	0.9931	0.978	0.9852	0.03	0.1	0.12
M5	697.4592	y = 655,508x − 2198	y = 939,291x − 66,379	y = 741,243x − 23,304	0.9864	0.969	0.9835	0.57	2.94	2.8
M6	682.4482	y = 13,396,606x + 350,014	y = 23,961,874x − 122,911	y = 14,079,951x − 586,077	0.996	0.9918	0.9952	0.08	0.06	0.1
M7	578.5152	y = 339,792x + 21,795	y = 516,253x + 73,837	y = 423,550x − 1278	0.9901	0.9937	0.9985	0.82	0.71	1.25

SO—sunflower oil; RO—rapeseed oil; SeO—sesame oil; asterix (*)—the value was not calculated.

**Table 3:** Occurrence of black seed oil, evening primrose oil, hemp oil, milk thistle oil, pumpkin seed oil, sunflower oil, and flaxseed oil metabolite markers in 7 commercial unrefined, cold-pressed vegetable oils blends, of two-, three- and four-component.

No.	*m/z*	Mix1	Mix2	Mix3	Mix4	Mix5	Mix6	Mix7
		Black seed oil compliance with labeling						
		2%	20%	0%	0%	0%	0%	4%
M1	166.1226	+	+	−	−	−	−	+
M2	328.1551	+	+	−	−	−	−	+
M3	342.1710	+	+	−	−	−	−	+
M4	313.1802	+	+	−	−	−	−	+
M5	238.0901	+	+	−	−	−	−	+
M6	131.0862	+	+	−	−	−	−	+
M7	385.2376	+	+	−	−	−	−	+
M8	404.2641	+	+	−	−	−	−	+
M9	677.3231	+	+	−	−	−	−	+
M10	635.3118	+	+	−	−	−	−	+
		Evening primrose oil compliance with labeling						
		0%	0%	3%	20%	0%	0%	5%
M1	469.3317	−	−	+	+	−	−	+
M2	455.3161	−	−	−	+	−	−	−
M3	409.2207	−	−	−	+	−	−	−
M4	492.3174	−	−	−	+	−	−	+
M5	634.4167	−	−	−	−	−	−	+
M6	720.4547	−	−	−	+	−	−	+
M7	577.39	−	−	+	+	−	−	+
M8	429.3741	−	−	−	+	−	−	+
		Hemp oil compliance with labeling						
		0%	0%	0%	0%	0%	5%	0%
M1	375.2178	−	−	−	−	−	+	−
M2	369.1343	−	−	−	−	−	−	−
M3	437.1965	−	−	−	−	−	−	−
M4	341.2123	−	−	−	−	−	+	−
M5	315.2329	−	−	−	−	−	+	−
M6	498.3586	−	−	−	−	−	+	−
M7	460.4163	−	−	−	−	−	−	−
		Milk thistle oil compliance with labeling						
		0%	0%	27%	0%	20%	0%	0%
M1	145.0764	−	−	+	−	+	−	−
M2	483.1296	−	−	+	−	+	−	−
M3	233.1545	−	−	+	−	+	−	−
M4	239.2013	−	−	+	−	+	−	−
		Pumpkin seed oil compliance with labeling						
		0%	0%	0%	0%	0%	10%	4%
M1	269.1904	−	−	−	−	−	+	+
M2	181.1231	−	−	−	−	−	+	−
M3	358.2389	−	−	−	−	−	+	+
M4	578.4219	−	−	−	−	−	+	+
M5	697.4592	−	−	−	−	−	+	+
M6	682.4482	−	−	−	−	−	+	+
M7	578.5152	−	−	−	−	−	−	−
		Sunflower oil compliance with labeling						
		0%	0%	0%	40%	0%	0%	0%
M1	231.1386	−	−	−	+	−	−	−
M2	217.0865	−	−	−	+	−	−	−
M3	345.0975	−	−	−	+	−	−	−
M4	203.1076	−	−	−	+	−	−	−
M5	301.2172	−	−	−	+	−	−	−
M6	392.2444	−	−	−	+	−	−	−
M7	420.2758	−	−	−	+	−	−	−
M8	432.2758	−	−	−	+	−	−	−
M9	434.2913	−	−	−	+	−	−	−
M10	576.3394	−	−	−	+	−	−	−
M11	590.3552	−	−	−	+	−	−	−
M12	604.3699	−	−	−	+	−	−	−
M13	618.3865	−	−	−	+	−	−	−
		Flax seed oil compliance with labeling						
		98%	80%	70%	40%	40%	85%	0%
M1	212.0823	+	+	+	+	+	+	−
M2	287.2016	+	+	+	+	+	+	−
M3	285.1853	+	+	+	+	+	+	−

## Data Availability

The data is available upon request.

## Supplementary Materials

The following supporting information can be downloaded at: https://www.mdpi.com/article/10.3390/molecules28124754/s1, Figure S1. LC–QTOF extracted ion chromatograms of oil metabolite markers in 7 commercial unrefined, cold-pressed vegetable oils blends, of two-, three- and four-component (Mix 1- Mix 7); Table S1: Preparation of binary oil mixtures; Table S2: Differences in the intensity of the oil markers in products from different producers and from different production batches, expressed as relative standard deviation of the peak areas of the extracted ion chromatograms (EIC); Table S3: LOD for sunflower, rapeseed and sesame specific metabolomic markers; Table S4: Composition of the commercial unrefined, cold- pressed oil blends products.

## Sample Availability

Samples of the compounds are not available from the authors.
