# Supplementary material for: LC–MS Metabolomic Profiling of Five Types of Unrefined, Cold-Pressed Seed Oils to Identify Markers to Determine Oil Authenticity and to Test for Oil Adulteration
Source: Molecules. 2023 Jun 14;28(12):4754. doi: 10.3390/molecules28124754 (PMC10305576; doi:10.3390/molecules28124754)
Supplement: Supplementary file 1 [file molecules-28-04754-s001.zip › molecules-2420319-supplementary.pdf]

SUPPLEMENTARY MATERIAL

LC–MS metabolomic profiling of five types of unrefined, cold-pressed seed oils to identify markers to determine oil authenticity and to test for oil adulteration

Agata Sumara[1], Anna Stachniuk[1], Alicja Trzpil[1], Adrian Bartoszek[1], Magdalena Montowska[2], Emilia Fornal[1*]

[1] Department of Bioanalytics, Medical University of Lublin, ul. Jaczewskiego 8b, 20-090 Lublin, Poland; agata.sumara@umlub.pl (A.S.); anna.stachniuk@umlub.pl (A.S.); alicja.trzpil@umlub.pl (A.T.); adrian.bartoszek@umlub.pl (A.B.)
[2] Department of Meat Technology, Poznan University of Life Sciences, ul. Wojska Polskiego 31, 60-624 Poznan, Poland; magdalena.montowska@up.poznan.pl
* Correspondence: emilia.fornal@umlub.pl

Table S1. Preparation of binary oil mixtures

OIL	5%	10%	30%	50%	70%	90%
**Black seed**<br>**Evening Primrose**<br>**Hemp**<br>**Milk Thistle**<br>**Pumpkin**	25 µl	50 µl	150 µl	250 µl	350 µl	450 µl
**Sunflower**<br>**Rapeseed**<br>**Sesame**	475 µl	450 µl	350 µl	250 µl	150 µl	50 µl

Table S2. Differences in the intensity of the oil markers in products from different producers and from different production batches, expressed as relative standard deviation of the peak areas of the extracted ion chromatograms (EIC).

**Oil**	**No**	**Observe *m/z***	**tR [min]**	**RSD [%]**
**Black seed**	M1	166.1226	2.6	36.48
	M2	328.1551	4.2	90.14
	M3	342.1710	5.1	64.15
	M4	313.1802	7.3	49.58
	M5	238.0901	7.9	90.70
	M6	131.0862	9.7	47.15
	M7	385.2376	11.4	84.36
	M8	404.2641	13.1	128.73
	M9	677.3231	19.1	78.66
	M10	635.3118	20.9	94.15
**Evening Primrose**	M1	469.3317	10.9	29.31
	M2	455.3161	11.8	30.38
	M3	409.2207	14.4	72.38
	M4	492.3174	16.5	78.06
	M5	634.4167	20.9	90.87
	M6	720.4547	21.1	92.00
	M7	577.39	22.9	25.71
	M8	429.3741	24.2	25.27
**Hemp seeds**	M1	375.2178	13.3	59.37
	M2	369.1343	14.4	45.96
	M3	437.1965	18.3	48.36
	M4	341.2123	18.6	55.57
	M5	315.2329	19.3	49.80
	M6	498.3586	22.6	57.13
	M7	460.4163	28.2	65.57
**Milk Thistle**	M1	145.0764	2.1	61.21
	M2	483.1296	8.8	67.97
	M3	233.1545	11.6	68.34
	M4	239.2013	12.1	103.14
**Pumpkin seed**	M1	269.1904	8.7	54.06
	M2	181.1231	17.9	63.41
	M3	358.2389	18.4	28.39
	M4	578.4219	19.6	14.72
	M5	697.4592	22.1	66.81
	M6	682.4482	25.1	29.66
	M7	578.5152	28.6	50.18

Table S3. LOD for sunflower, rapeseed and sesame specific metabolomic markers.

OIL				Black seed	Hemp	Evening Primrose	Milk Thistle	Pumpkin seed
				LOD	LOD	LOD	LOD	LOD
**Sunflower**	1	231.1386	10.03 ± 0.18	25.00	3.49	*	25.00	*
	2	217.0865	11.31 ± 0.25	2.91	0.38	8.11	12.00	3.49
	3	345.0975	11.77 ± 0.25	14.29	*	18.75	*	*
	4	203.1076	12.76 ± 0.24	3.30	0.40	0.71	2.40	0.90
	5	301.2172	13.15 ± 0.2	14.29	16.67	15.79	12.00	13.04
	6	392.2444	14.14 ± 0.26	0.52	0.42	1.60	2.83	1.35
	7	420.2758	16.76 ± 0.3	9.09	0.28	0.66	3.57	4.48
	8	432.2758	17.47 ± 0.28	6.52	0.52	1.60	2.83	2.04
	9	434.2913	17.98 ± 0.33	4.69	0.20	4.92	2.59	2.13
	10	576.3394	19.44 ± 0.26	*	*	*	21.43	25.00
	11	590.3552	20.23 ± 0.31	14.29	1.96	1.62	3.80	2.63
	12	604.3699	20.97 ± 0.3	8.33	2.73	5.77	4.11	5.66
	13	618.3865	21.74 ± 0.26	*	0.80	7.14	8.33	8.33
**Rapeseed**	14	162.0557	6.01 ± 0.28	1.29	1.23	1.66	2.17	4.55
	15	225.1488	6.44 ± 0.16	10.00	10.71	10.71	9.38	17.65
	16	288.1237	10.05 ± 0.13	1.26	0.48	5.45	3.41	5.36
	17	467.3510	20.42 ± 0.3	*	1.09	3.37	9.09	13.04
	18	483.3824	22.01 ± 0.18	*	14.29	5.08	*	*
	19	379.3365	24.26 ± 0.34	*	0.67	16.67	*	14.29
	20	413.3429	24.81 ± 0.34	0.68	0.15	1.68	4.35	3.30
	21	773.5184	27.95 ± 0.7	*	0.72	6.82	13.04	20.00
**Sesame**	22	373.1286	8.71 ± 0.15	5.66	3.49	12.00	13.64	8.57
	23	371.1136	10.6 ± 0.19	0.15	0.12	0.59	1.52	0.55
	24	299.2592	12.88 ± 0.07	0.62	0.85	1.38	4.69	2.83
	25	337.1082	13.66 ± 0.22	0.23	0.11	0.63	1.01	3.09
	26	173.0604	14.53 ± 0.23	0.30	0.11	3.03	1.25	2.63

LOD - Limit of detection; asterix (*) - value no calculated; metabolomic markers of sunflower, rapeseed and sesame oil were as reported previously by Sumara et al. 2023

Table S4. Composition of the commercial unrefined, cold- pressed oil blends products

**Name**	**No.**	**Oil composition**			
Oil for children	Mix 1	98% Flax seed	2% Black seed		
Oil for the heart	Mix 2	80% Flax seed	20% Black seed		
Oil for women	Mix 3	70% Flax seed	27% Milk thistle	3% Evening primrose	
Oil for mothers	Mix 4	40% Flax seed	40% Sunflower	20% Evening primrose	
Oil for elderly	Mix 5	40% Flax seed	40% Camelina	20% Milk Thistle	
Oil for men	Mix 6	85% Flax seed	10% Pumpkin seed	5% Hemp	
Immuno oil	Mix 7	87% Camelina	5% Evening primrose	4% Pumpkin seed	4% Black seed

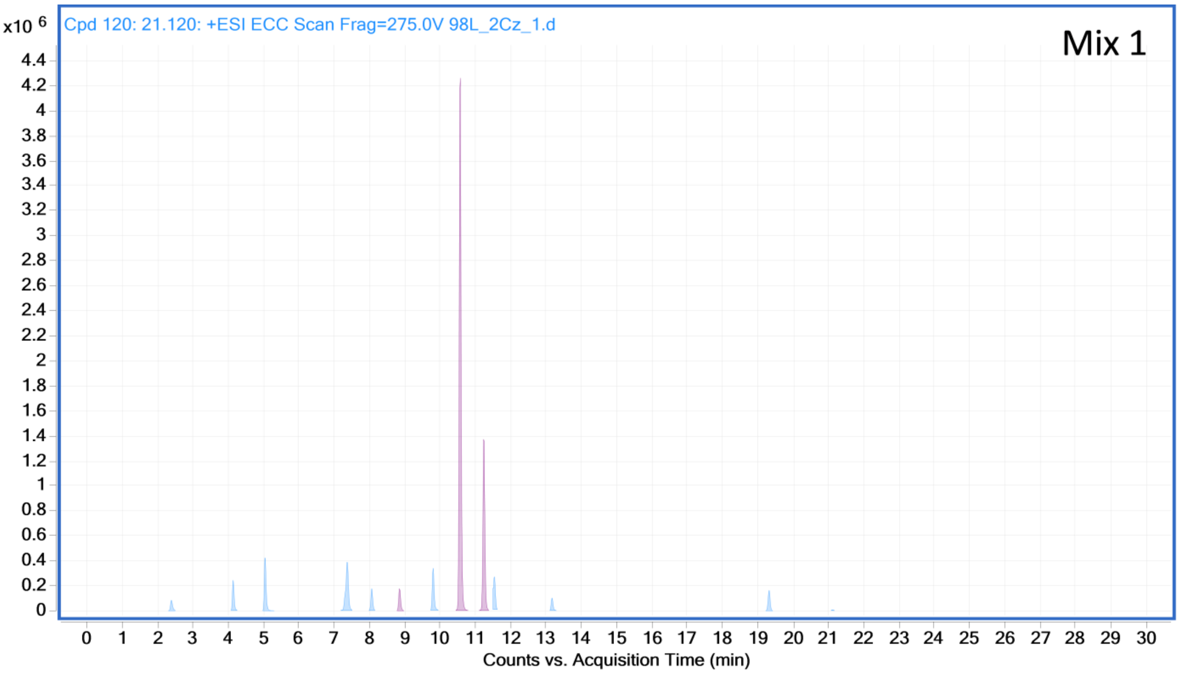

x10 6
Cpd 120: 21.120: +ESI ECC Scan Frag=275.0V 98L_2Cz_1.d
Mix 1
Counts vs. Acquisition Time (min)

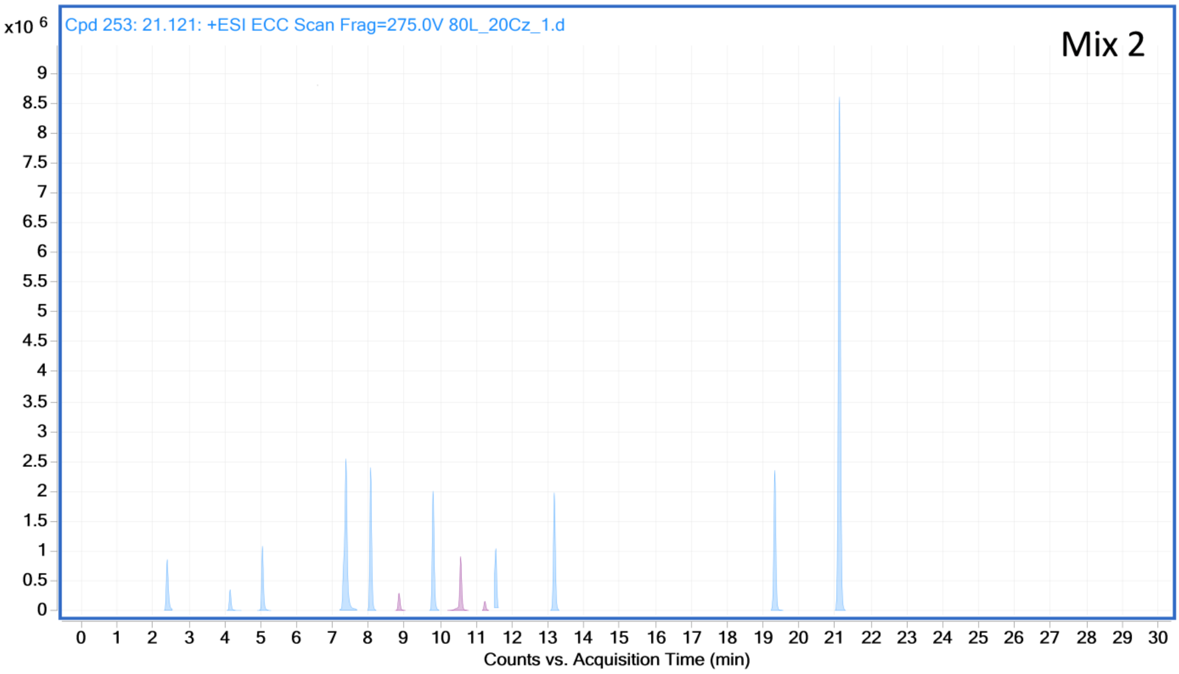

x10 6
Cpd 253: 21.121: +ESI ECC Scan Frag=275.0V 80L_20Cz_1.d
Mix 2
Counts vs. Acquisition Time (min)

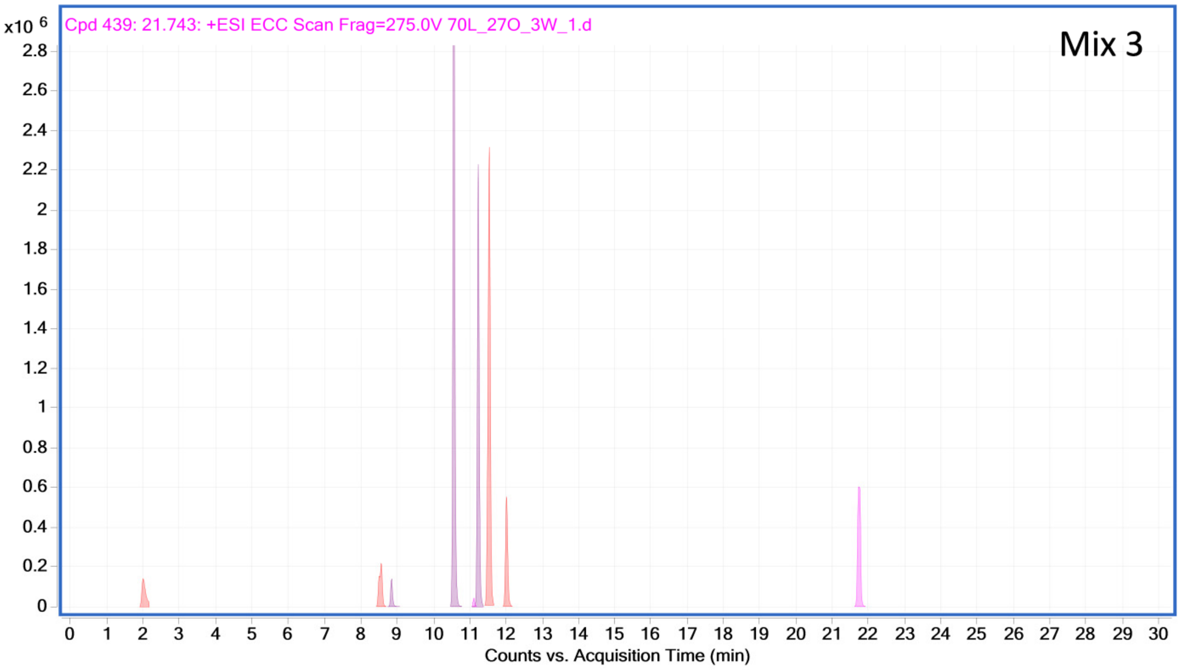
x10 6
Cpd 439: 21.743: +ESI ECC Scan Frag=275.0V 70L_27O_3W_1.d
Mix 3
Counts vs. Acquisition Time (min)

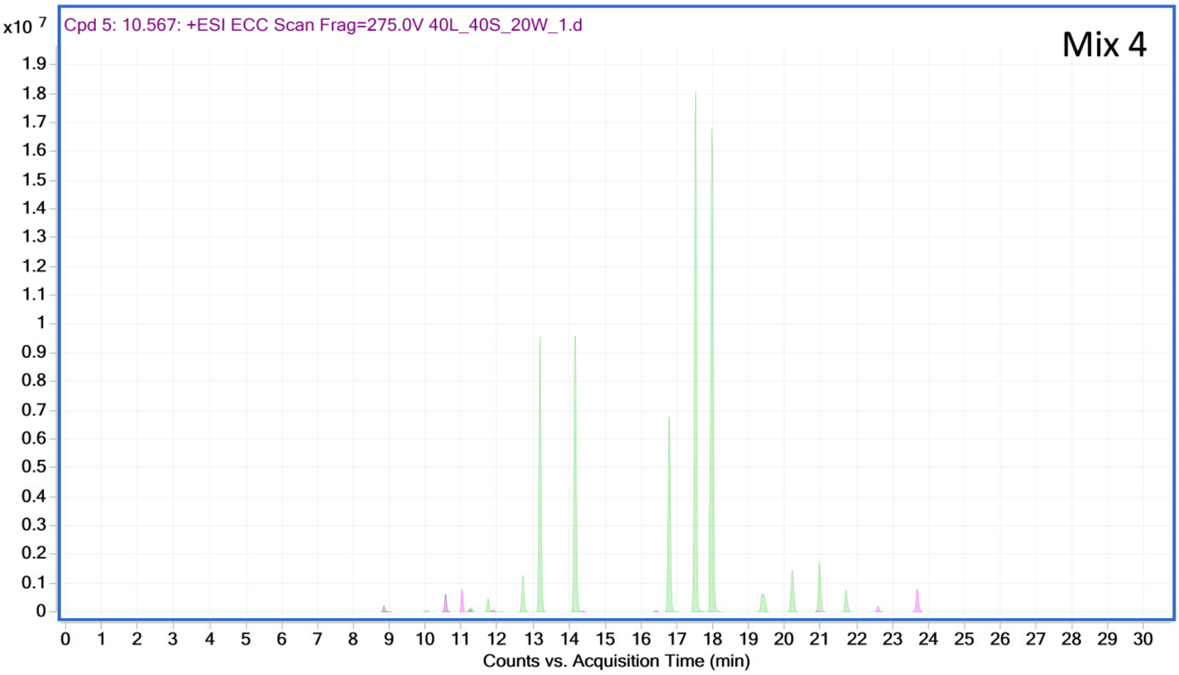
x10 7
Cpd 5: 10.567: +ESI ECC Scan Frag=275.0V 40L_40S_20W_1.d
Mix 4
Counts vs. Acquisition Time (min)

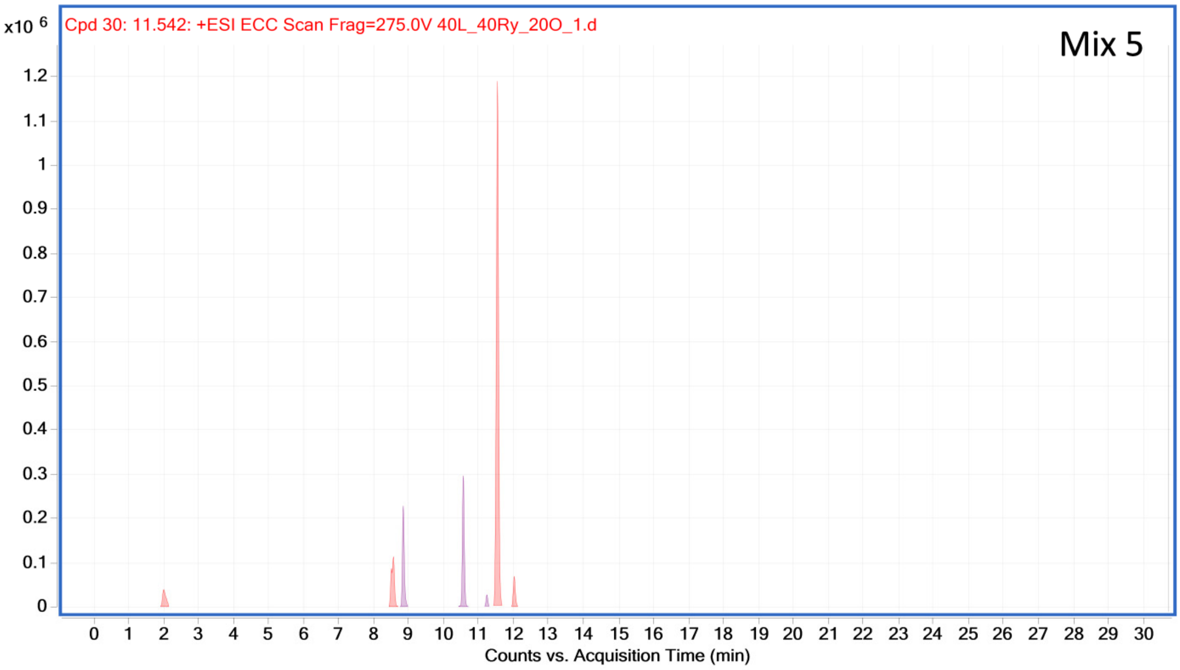

x10 6
Cpd 30: 11.542: +ESI ECC Scan Frag=275.0V 40L_40Ry_20O_1.d
Mix 5
1.2
1.1
1
0.9
0.8
0.7
0.6
0.5
0.4
0.3
0.2
0.1
0
0 1 2 3 4 5 6 7 8 9 10 11 12 13 14 15 16 17 18 19 20 21 22 23 24 25 26 27 28 29 30
Counts vs. Acquisition Time (min)

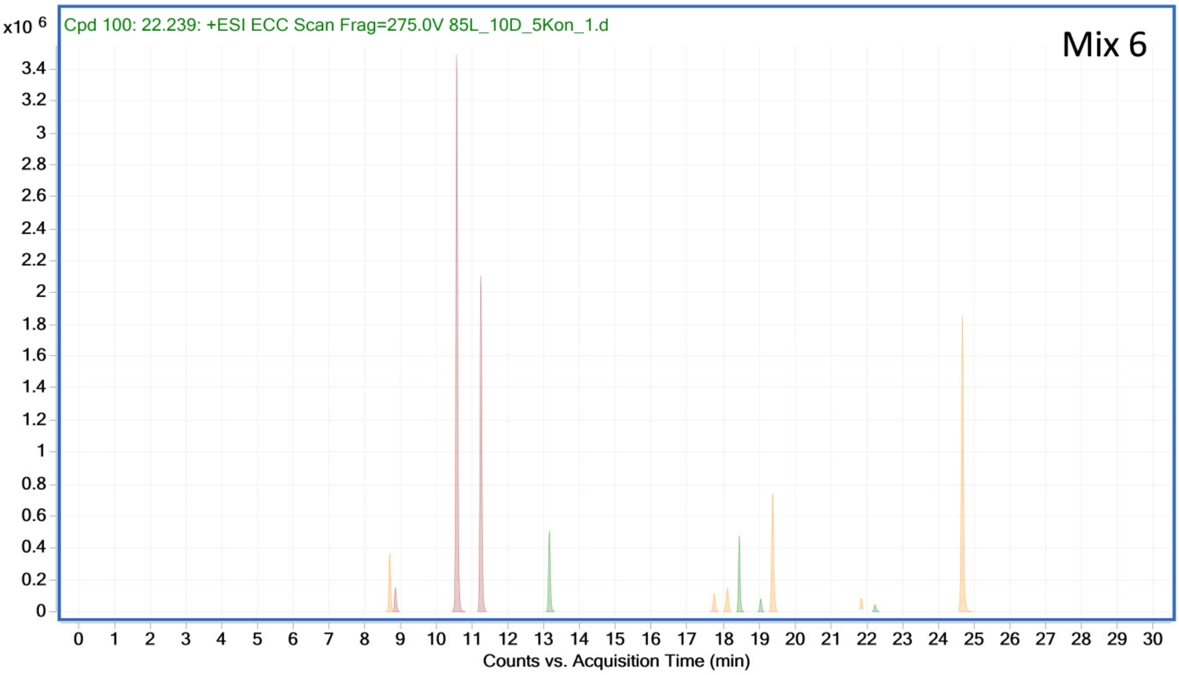

x10 6
Cpd 100: 22.239: +ESI ECC Scan Frag=275.0V 85L_10D_5Kon_1.d
Mix 6
3.4
3.2
3
2.8
2.6
2.4
2.2
2
1.8
1.6
1.4
1.2
1
0.8
0.6
0.4
0.2
0
0 1 2 3 4 5 6 7 8 9 10 11 12 13 14 15 16 17 18 19 20 21 22 23 24 25 26 27 28 29 30
Counts vs. Acquisition Time (min)

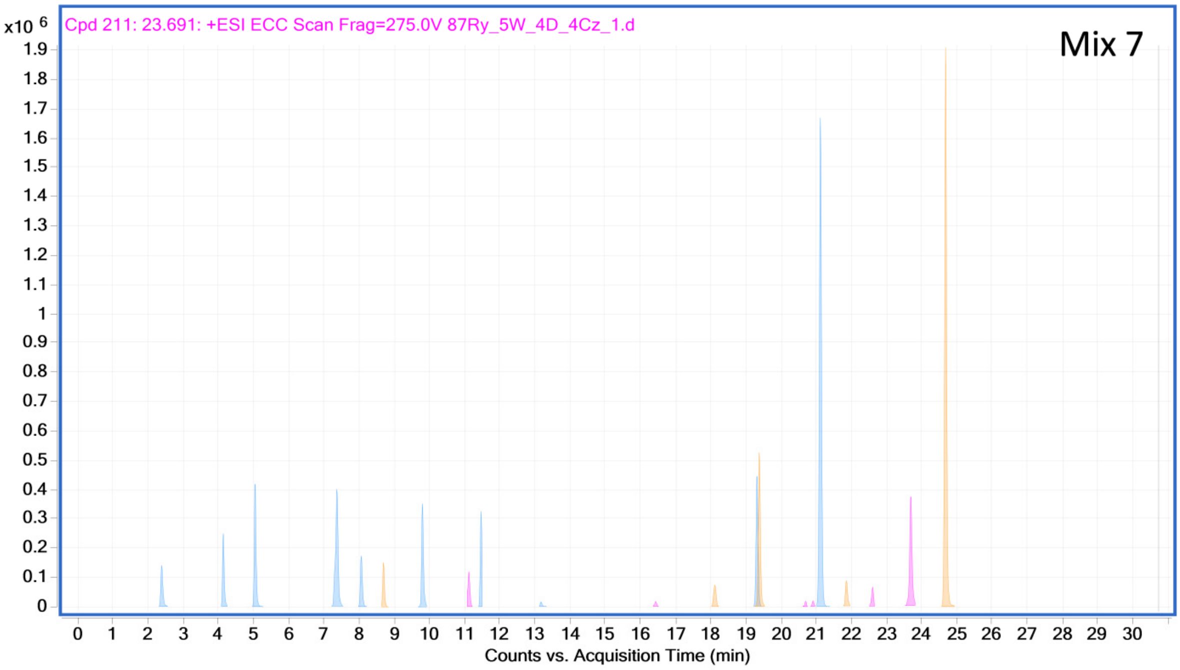

Figure S1. LC–QTOF extracted ion chromatograms of oil metabolite markers in 7 commercial unrefined, cold-pressed vegetable oils blends, of two-, three- and four-component (Mix 1- Mix 7). Flax seed oil markers - purple, milk thistle oil markers - red, evening primrose oil markers - pink, sunflower oil markers - light green, black seed oil markers- blue, pumpkin seed oil markers- orange, hemp oil markers- green. All details for oil specific markers are presented in Fig. S1 are given in Table 1 and Table S3
